# Supplementary material for: A Critical View of the Application of the APEX Software (Aqueous Photochemistry of Environmentally-Occurring Xenobiotics) to Predict Photoreaction Kinetics in Surface Freshwaters
Source: Molecules. 2019 Dec 18;25(1):9. doi: 10.3390/molecules25010009 (PMC7017383; doi:10.3390/molecules25010009)
Supplement: Supplementary file 1 [file molecules-25-00009-s001.zip › 3.2.4_gcc-4.4.0.zip!/3.2.4_gcc-4.4.0/3.2.4_gcc-4.4.0/doc/octave/html/interpreter/The-_003ccode_003ebreak_003c_002fcode_003e-Statement.html]

The @code{break} Statement - Untitled


Next: The <code>continue</code> Statement,
Previous: The <code>for</code> Statement,
Up: Statements


---

### 10.6 The `break` Statement

The `break` statement jumps out of the innermost `for` or
`while` loop that encloses it. The `break` statement may only
be used within the body of a loop. The following example finds the
smallest divisor of a given integer, and also identifies prime numbers:

```
     num = 103;
     div = 2;
     while (div*div <= num)
       if (rem (num, div) == 0)
         break;
       endif
       div++;
     endwhile
     if (rem (num, div) == 0)
       printf ("Smallest divisor of %d is %d\n", num, div)
     else
       printf ("%d is prime\n", num);
     endif
```

When the remainder is zero in the first `while` statement, Octave
immediately breaks out of the loop. This means that Octave
proceeds immediately to the statement following the loop and continues
processing. (This is very different from the `exit` statement
which stops the entire Octave program.)

Here is another program equivalent to the previous one. It illustrates
how the condition of a `while` statement could just as well
be replaced with a `break` inside an `if`:

```
     num = 103;
     div = 2;
     while (1)
       if (rem (num, div) == 0)
         printf ("Smallest divisor of %d is %d\n", num, div);
         break;
       endif
       div++;
       if (div*div > num)
         printf ("%d is prime\n", num);
         break;
       endif
     endwhile
```
